# Accessory Head of Sternocleidomastoid Muscle in Indian Cadavers: A Report of Three Cases

**DOI:** 10.7759/cureus.63547

**Published:** 2024-06-30

**Authors:** Sulochana Sakthivel, Nithya Dhakshnamoorthy, Sankara Narayanan G

**Affiliations:** 1 Anatomy, Jawaharlal Institute of Postgraduate Medical Education and Research, Puducherry, IND; 2 Anatomy, Saveetha Medical College and Hospital, Chennai, IND

**Keywords:** venous catheterization, torticollis, clavicle, sternocleidomastoid muscle flap, brachial plexus block

## Abstract

During dissection sessions for undergraduate students, the unilateral accessory clavicular head of the sternocleidomastoid muscle was observed in three cadavers. These accessory heads extended from the middle third of the clavicle and joined the sternocleidomastoid muscle in the middle third. The variations in the sternocleidomastoid muscle may be attributed to abnormal mesodermal splitting or fusion failure during the development of the post-sixth branchial arch. Anomalies of the sternocleidomastoid may be misdiagnosed as cervical dystonia, fibromatosis colli, or muscular spasm. In rare cases, an accessory head could result in torticollis in adults. These anomalies warrant particular attention during interventional procedures conducted by anesthesiologists. The internal jugular vein is accessed at the lesser supraclavicular fossa for cannulation during central venous access and temporary hemodialysis. Variations in its anatomy can pose challenges during these procedures. Moreover, the clavicular head may be utilized for muscle flaps in the upper neck and occipital regions.

## Introduction

The sternocleidomastoid (SCM) is the key muscle of the cervical region, integrated with proprioception and cranial and autonomic nerves to execute protective, involuntary head movements. The SCM muscle originates from the sternum and clavicle, extending upward, backward, and laterally to insert on the mastoid bone and superior nuchal line [1]. The mastoid process, a protrusion of the temporal bone, appears to protrude downward behind the auricle by the third year after birth through traction of the SCM muscle [2]. The SCM muscle forms the anterior boundary of the lesser supraclavicular triangle, with the jugular vein located within this triangle. Medical and surgical Interventions like central venous catheterization, supraclavicular lymph node biopsy, and supraclavicular brachial plexus block are often performed in this area and may be impacted by the presence of accessory heads of the SCM [3,4]. Such anatomical variations can lead to potential complications such as pneumothorax, air embolism, puncture of the subclavian artery, Horner syndrome, and phrenic nerve damage [5].

The primary causes of torticollis or wryneck are attributed to muscular (SCM anomalies), ocular (nystagmus), and osseous (cervical or thoracic dysgenesis) factors. Muscular torticollis is often associated with either ocular or osseous causes. [6]. Congenital SCM anomalies, SCM neoplasms, or peripartum SCM injury underlie the development of torticollis [7]. Torticollis represents a rare congenital anomaly, with an incidence ranging from 0.2% to 2% [8]. The diagnosis and management of this condition pose significant challenges due to varied pathologies (congenital or acquired) and a limited spectrum or lack of clinical manifestations (head tilt, neurological pain, restricted range of movement) especially, if it is congenital [9].

Awareness of the developmental anomalies of SCM is crucial for anaesthesiologists and surgeons when approaching the lesser supraclavicular triangle clinically. Such awareness can help improve their clinical approach and prevent iatrogenic complications. We are reporting three cases of unilateral accessory heads of the SCM found in Indian cadavers.

## Case presentation

During the period of 2021 to 2023, 24 cadavers of Indian origin were utilized for dissection classes for undergraduate students in the Department of Anatomy. These cadavers were procured through the Body Donation Program and have been approved for medical education and research. In three cadavers (12.5%), the accessory head of the SCM was encountered. The length of the SCM muscle was measured on the anterior and posterior borders using the digital vernier caliper with a sensitivity of 0.1 mm.

Case 1

**Figure 1:**
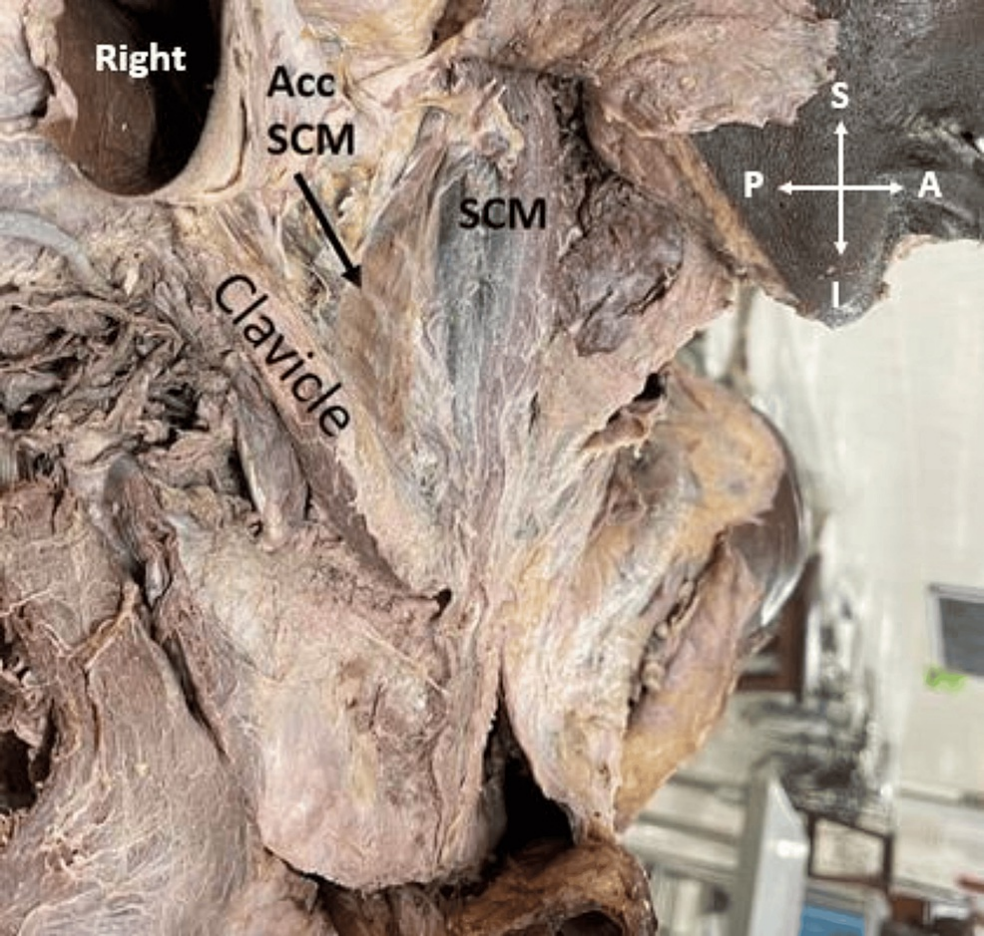
Thin, flattened, and triangular-shaped accessory head of the SCM muscle extending from the middle third of the clavicle to the SCM on the right side. Acc: accessory; SCM: sternocleidomastoid; S: superior; I: inferior; A: anterior; P: posterior

In a 45-year-old male cadaver, the accessory head of SCM was observed on the right side. The thin, flattened triangular-shaped accessory head of the SCM muscle extended from the middle third of the clavicle to the middle of the SCM. The muscle length was measured as 5.33 cm on the anterior border, and 4.36 cm on the posterior border. The lower attachment of the muscle on the clavicle measured 1.7 cm (Figure 1).

Case 2

**Figure 2:**
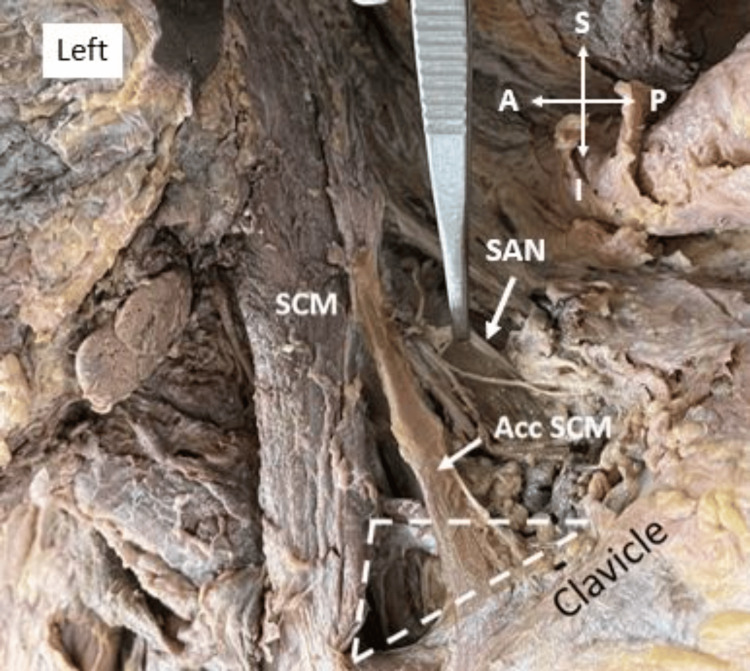
A thin, strap-like, triangular-shaped accessory head of SCM muscle extends from the middle third of the clavicle to the SCM on the left side. The supraclavicular triangle is shown in a dotted line. Acc: accessory; SCM: sternocleidomastoid; SAN: spinal accessory nerve; S: superior; I: inferior; A: anterior; P: posterior

In the second case, a triangular accessory head of the SCM muscle was identified on the left side of a 60-year-old male cadaver. The accessory head originated from the middle third of the clavicle and merged with the SCM muscle. The anterior border of the muscle was 6.93 cm long, and the posterior border was 7.62 cm. The width of the muscle attachment on the clavicle was measured as 2.15 cm (Figure 2).

Case 3

**Figure 3:**
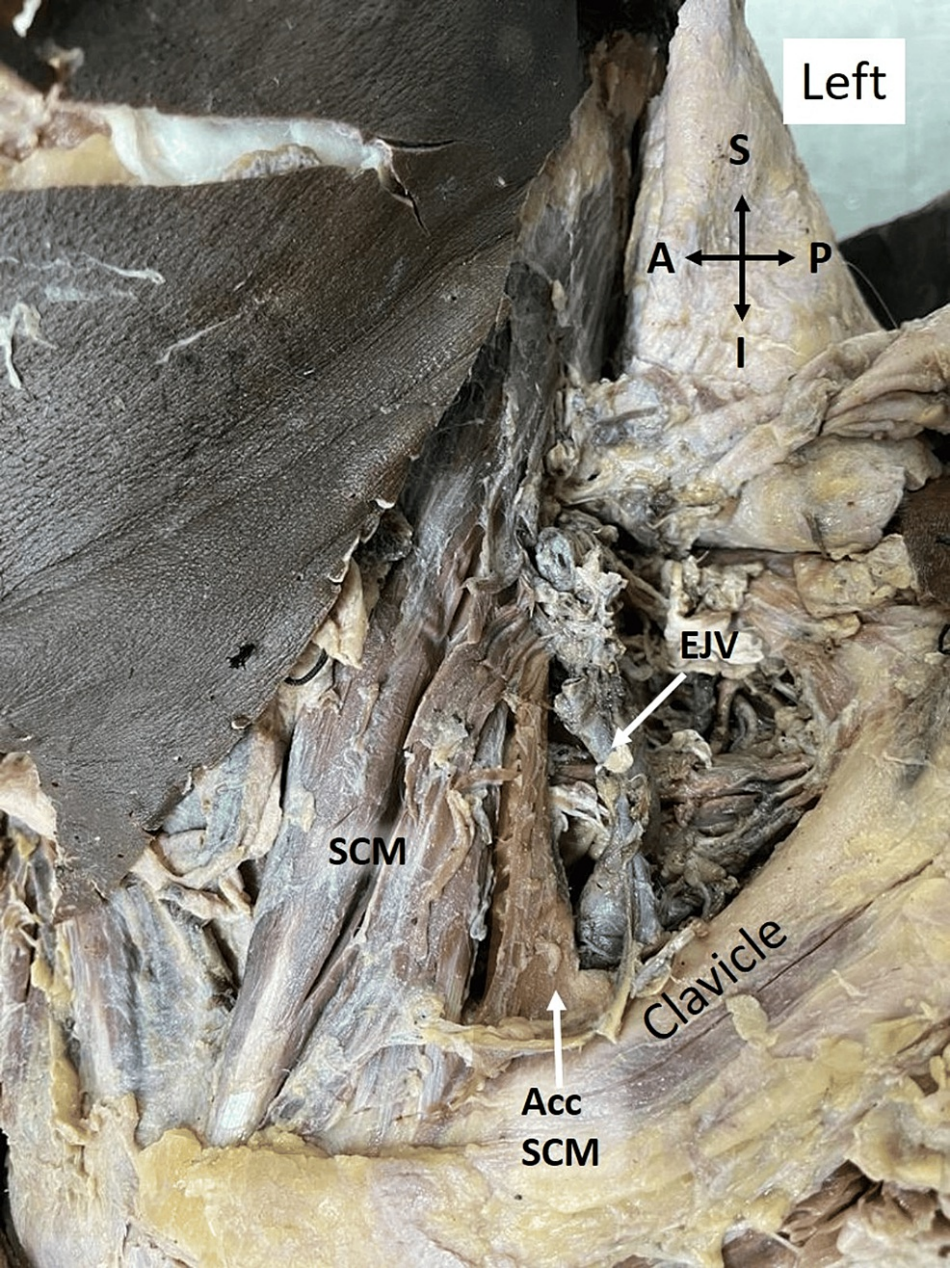
Attachment of the thick accessory head of SCM at the junction of the medial and middle thirds of the left clavicle. Acc: accessory; SCM: sternocleidomastoid; EJV: external jugular vein; S: superior; I: inferior; A: anterior; P: posterior

In the third case, the accessory head was observed in a 68-year-old male cadaver on the left side. In this instance, the clavicular attachment of SCM and its thick accessory head were dissimilar from the previous cases. The accessory head originated from the junction of the medial and middle third of the clavicle positioning the muscle closer to the main SCM. The anterior border of the muscle measured 6.71 cm, with the posterior border spanning 6.43 cm in length. The width of the attachment of the muscle on the clavicle was 2.27 cm (Figure 3). The spinal accessory nerve innervated the accessory head of SCM in all three cadavers.

## Discussion

Abnormal mesodermal splitting or fusion failure during the development of the post-sixth branchial arch results in the formation of accessory heads of SCM. The defective retinoic acid response element (RARE) in the *Hox* gene and the impaired interaction with the *DLX* gene are the primary factors contributing to this developmental anomaly [10]. Phylogenetically, SCM and trapezius originate from a muscle called cucullaris. During mammalian evolution, the muscle mass splits into two parts forming the SCM and trapezius [11]. The developing clavicle, scapula, and head of the humerus provide the major drive for posterolateral traction. This leads to the separation of SCM and trapezius in humans and is completed at the fifth week of gestation [12]. Singh et al. reported a case of an absent posterior triangle due to the presence of a muscle sheet extending between the SCM and trapezius, which obscured the posterior triangle with a small oval gap observed postero-superiorly on the left posterior triangle in a 60-year-old male Indian cadaver [13]. The present variation could be due to the persistence of muscle slips between the SCM and trapezius.

Congenital muscular torticollis caused by accessory heads of SCM leads to a restricted range of head and neck movement. It may also be associated with congenital osseous or ocular torticollis [6]. Multiple factors can contribute to torticollis, including damage to the SCM during delivery, tumors or pseudotumors involving the SCM, and abnormal postural damage [14]. Generally, mild torticollis is more asymptomatic than moderate to severe cases. However, any unnoticed torticollis could be associated with facial and skeletal deformities. [15].

Even though the studies regarding the accessory head of the SCM are limited in the literature, several cases have been reported. Mansoor et al. reported a case of an accessory head in the right SCM with restricted movement on the right side [16]. Obajeun et al. documented a case of neglected left-sided torticollis in an infant with a complex cervical spine deformity, characterized by a mass and shortening of the left SCM due to injury during labor [17]. Kim et al. reported two cases of secondary congenital muscular torticollis with the primary cause as the congenital osseous (T1 hemivertebra) and the congenital ocular torticollis (agenesis of the left trochlear nerve) in a one-month and a three-month-old infant, respectively [6]. Failure to recognize and address torticollis may be misdiagnosed as cervical dystonia and lead to permanent skeletal deformities in the cervical region [18]. In our report, the unilateral accessory head of SCM was observed in three cadavers; one on the right side and two on the left side. In all cases, the accessory head originated from the clavicle and was inserted in the middle third of the SCM.

Maxilla-facial surgeries require SCM muscle flap to reconstruct the face, cheek, mandible, floor of the mouth, and pharyngeal and laryngeal complexes [19]. SCM flap is harvested to prevent Frey's syndrome. Sanabria et al. found improvement in signs and incidence of Frey's syndrome, though the symptoms remain the same [20]. In addition, the SCM flap is also harvested to close the tracheoesophageal fistula developed as the result of treating laryngeal cancer [21]. Hence, harvesting the SCM muscle flap may be challenging and complicated in the case of SCM with an accessory head.

## Conclusions

Sternocleidomastoid is a crucial muscle in the cervical region, and its variant anatomy holds considerable significance and poses challenges in various medical interventions such as flap reconstructions using SCM, nerve block at nerve point, surgical release of SCM in torticollis, supraclavicular brachial plexus block and jugular venous catheterization. Awareness of the SCM variation will provide a wide range of options to the maxilla-facial surgeons during facial reconstructive surgeries using SCM flap and in the differential diagnosis of torticollis.
